# Dihydromyricetin May Attenuate Skin Aging as a RAGE Inhibitor

**DOI:** 10.3390/nu17111862

**Published:** 2025-05-29

**Authors:** Fei Wang, Yuanzhi Jian, Fangzhi Xia, Liangchun Kuo, Junbo Wang

**Affiliations:** 1 Department of Nutrition and Food Hygiene, School of Public Health, Peking University, Beijing 100191, China; wangfei.duzhuo@163.com (F.W.); 2211210080@stu.pku.edu.cn (F.X.); kuo_liangchun@bjmu.edu.cn (L.K.); 2 Jinan Municipal Center for Disease Control and Prevention, Jinan 250021, China; 1610306107@pku.edu.cn; 3 Beijing Key Laboratory of Toxicological Research and Risk Assessment for Food Safety, Peking University, Beijing 100191, China

**Keywords:** dihydromyricetin, skin aging, advanced glycation end products, AGE-RAGE signaling, transcriptome

## Abstract

**Background/Objectives**: Dihydromyricetin (DHM), a flavonoid with abundant natural sources, potent bioactivity, and high safety, holds promise for translational applications, particularly in mitigating skin aging. However, its role and underlying mechanisms in counteracting skin aging induced by advanced glycation end products (AGEs) remain unclear. **Methods**: Eight-week-old male Sprague-Dawley (SD) rats were subcutaneously injected with 500 mg/kg D-galactose and administered DHM via gavage for 11 weeks. Additionally, senescent human skin fibroblasts (HFF-1) induced by AGEs were used for further investigation. **Results**: DHM treatment significantly alleviated D-galactose-induced skin aging in rats, with the most pronounced effects observed in the moderate-dose group (100 mg/kg). Compared to the aging group, DHM enhanced skin elasticity and preserved collagen levels. Moreover, DHM promoted cell proliferation in the skin. Further studies on AGE-induced senescent fibroblasts revealed that DHM markedly reduced multiple senescence-associated markers and stimulated cell proliferation by approximately a 1.5-fold increase. Transcriptomic analysis indicated that DHM upregulated genes related to the cell cycle and DNA repair while suppressing AGE-RAGE signaling and its downstream pathways. Notably, DHM downregulated *AGER*, the gene encoding the receptor for AGEs (RAGE). Molecular docking analysis demonstrated that DHM shares a binding site with other known RAGE inhibitors. Surface plasmon resonance (SPR) analysis further confirmed the high binding affinity of DHM to RAGE (K_D_ = 28.7 μM), which was stronger and more stable than that of FPS-ZM1 (K_D_ = 40.7 μM). **Conclusions**: DHM may attenuate glycation-induced skin aging in rats by functioning as a RAGE inhibitor, thereby suppressing AGE-RAGE signaling, delaying cellular senescence, and promoting cell proliferation.

## 1. Introduction

Population aging is an inevitable global trend. According to the World Population Prospects 2024 report released by the United Nations, the proportion of individuals aged ≥65 years is projected to rise rapidly, potentially reaching 33% of the global population by 2054. Age-related functional decline and the rising prevalence of chronic diseases pose significant threats to human health, substantially increasing the global disease burden. Therefore, strategies aimed at delaying aging and promoting “healthy aging” have emerged as critical topics in 21st-century health research.

Skin aging is often one of the earliest visible manifestations of systemic aging processes. As the largest organ in the human body, the skin serves as the primary physical barrier against external stressors, such as ultraviolet (UV) radiation and pathogens. Age-related skin changes primarily include wrinkle formation, pigmentation, loss of elasticity, and thinning of the epidermis [1]. These alterations are accompanied by changes in collagen structure and composition, remodeling of elastic fibers, and a decline in hyaluronic acid-binding proteins, resulting in morphological and physiological changes. Such changes compromise the structural integrity and functional capacity of the skin, increasing susceptibility to dermatological disorders and posing significant health risks. Skin aging not only elevates the risk of skin-related diseases and impairs immune function [2,3] but is also closely correlated with systemic aging processes [4,5,6,7].

Factors contributing to skin aging are generally categorized as intrinsic or extrinsic [8,9,10]. These factors trigger collagen and elastin degradation, increase matrix metalloproteinase (MMP) levels, induce excessive production of advanced glycation end products (AGEs), and promote inflammation, thereby initiating or accelerating the aging process. Among these pathological and physiological processes, AGEs have attracted considerable research attention. AGEs are formed through the non-enzymatic glycation of macromolecules (such as proteins, lipids, and nucleic acids) by glucose or other reducing sugars in a reaction commonly known as the “Maillard reaction” [11]. In the late 1980s, Monnier et al. proposed the Maillard aging theory, suggesting that the gradual and persistent accumulation of AGEs is a major contributor to aging [12,13]. Numerous studies have confirmed the relationship between AGEs and age-associated diseases, linking AGEs to diabetes, cardiovascular diseases, neurodegenerative disorders, kidney disease, and skin-related conditions [11,14,15,16,17]. In skin tissue, AGEs can damage both the epidermis and dermis, affecting keratinocytes and melanocytes in the epidermis, fibroblasts in the dermis, and components of the extracellular matrix (ECM) in both layers [18,19]. AGEs accelerate skin aging via multiple mechanisms. They impair skin structure by forming cross-links with skin proteins and binding to their specific receptor, RAGE, thereby activating pro-inflammatory pathways. As a result, recent studies have identified AGEs as key mediators of skin aging and potential therapeutic targets for anti-aging interventions.

In recent years, phytochemicals have garnered increasing attention as promising agents for combating skin aging. These compounds offer potential nutritional and medicinal benefits with minimal or no side effects. Dihydromyricetin (DHM), a flavonoid with high bioactivity and safety, is widely distributed in plants. The content of DHM in vine tea can reach 30–40% [20], with additional sources including grapes, mulberries, ginkgo, *Hovenia acerba* seeds, and *Aquilaria sinensis* leaves [21,22,23], highlighting its considerable application potential. Recent studies have demonstrated the beneficial effects of DHM on aging and age-related diseases. Fan et al. [24] reported that dietary supplementation with 40 μM DHM extended the median lifespan of *Drosophilia* by 16.07%. In models of neurodegenerative disease, oral administration of DHM improved object recognition memory and spatial discrimination abilities in aging mice induced by D-galactose while also repairing histological damage, such as neuronal degeneration and fibrous tangle formation [25]. DHM treatment downregulated the expression of senescence-associated markers and ameliorated cognitive impairment in aged mice [26]. DHM has also shown promise in improving skin aging. Topical application of DHM in human subjects exhibited a skin-rejuvenating effect by altering UV-induced age-related methylation patterns [27]. In vitro, DHM reduced oxidative stress and inflammation induced by UVA radiation in human keratinocyte cells [28]. Additionally, dietary supplementation with 0.7% DHM alleviated UVB-induced skin lesions (e.g., redness, wrinkles, and sore ooze), with enhanced effects observed when combined with ellagic acid [29].

Although previous studies have reported the beneficial effects of DHM in improving skin aging, most have focused on UV-induced photoaging, with limited research addressing AGE-related or other intrinsic factors of skin aging. The role and mechanisms of DHM in attenuating AGE-induced skin aging remain unclear, however. Compared to UV-induced photoaging, AGE-related skin aging involves more complex pathogenic mechanisms and has broader systemic implications. Moreover, few effective agents target AGEs, underscoring the need for further research to develop prevention and treatment strategies. Therefore, this study investigated the effects of DHM on AGE-induced skin aging using a glycation-induced aging rat model and explored its anti-aging mechanisms using a cellular model of AGE-induced senescence.

## 2. Materials and Methods

### 2.1. Animals and Treatments

Forty 7-week-old male Sprague-Dawley (SD) rats were obtained from the Department of Laboratory Animal Science, Peking University Health Science Center. The animals were housed in a specific pathogen-free (SPF) facility under a 12:12 h light/dark cycle, with the temperature maintained at 24–26 °C and relative humidity at 50–60%. The rats were given ad libitum access to standard laboratory chow pellets and clean drinking water.

Skin aging was induced using D-galactose, as described in previous studies using SD rats [30,31]. After a one-week acclimation period, the rats were randomly divided into five groups (*n* = 8 per group): (1) Normal control (NC): subcutaneous saline injection and oral gavage with saline; (2) Aging group (SC): subcutaneous injection of 500 mg/kg D-galactose (purity ≥ 99%; Sigma, St. Louis, MO, USA) to induce skin aging and oral gavage with saline containing 0.1% DMSO (DHM solvent); (3) Low-dose DHM group (L): subcutaneous injection of 500 mg/kg D-galactose and oral administration of 50 mg/kg DHM (purity ≥ 98% by HPLC; Chengdu HerbSubstance Co., Ltd., Chengdu, China); (4) Moderate-dose DHM group (M): subcutaneous injection of 500 mg/kg D-galactose and oral administration of 100 mg/kg DHM; (5) High-dose DHM group (H): subcutaneous injection of 500 mg/kg D-galactose and oral administration of 300 mg/kg DHM. D-galactose was administered once daily for six days per week in all relevant groups. Saline or DHM treatment was administered once daily. An electric baby hair clipper was used to shave the rats for skin texture and wrinkle assessments.

After 11 weeks of treatment, the rats were fasted overnight and euthanized under anesthesia. Dorsal skin and serum samples were collected and stored at −80 °C until analysis. All animal protocols and procedures were approved by the Institutional Animal Care and Use Commission of Peking University (No: LA2022393).

### 2.2. Evaluation of Skin Wrinkles, Elasticity and Hydration

At the end of the 11-week treatment period, skin wrinkle formation, elasticity, and hydration were assessed using the DermaLab^®^ Combo3 system (Cortex Technology ApS, Hadsund, Denmark), which was equipped with multiple specialized probes. Specifically, a derma scope camera probe was used to evaluate fine skin features, the elasticity probe was used to measure skin elasticity, and the hydration pin probe was used to assess skin moisture levels. Prior to the assessment, hair was removed using an electric clipper followed by a depilatory agent (Veet Hair Removal Cream, Slough, UK) to ensure accurate measurements.

### 2.3. Histological Analysis

A 1 cm × 1 cm section of dorsal skin tissue was excised and immediately fixed in 4% paraformaldehyde (PFA). After 24 h, the tissues were embedded in paraffin and sectioned into 6 μm thick slices. Following deparaffinization, the slices were stained with hematoxylin and eosin (H&E) and Masson’s trichrome.

### 2.4. Immunohistochemical Staining

Immunohistochemical staining was performed to detect the expression of Ki67 and Col1a1 in the rat skin tissues. Briefly, after deparaffinization and antigen retrieval, the tissue sections were incubated overnight at 4 °C with primary antibodies against Ki67 (1:200, ab16667, Abcam, Cambridge, UK) or Col1a1 (1:200, #3033, Cell Signaling Technology, Danvers, MA, USA), followed by incubation with an HRP-conjugated secondary antibody. Images were captured using a microscope (Olympus, Tokyo, Japan).

### 2.5. Cell Culture and Treatments

The human skin fibroblast cell line HFF-1 was obtained from MeisenCTCC (Zhejiang Meisen Cell Technology Co., Ltd., Jinhua, China). Cells were cultured in Dulbecco’s Modified Eagle Medium (DMEM; Gibco, Grand Island, NY, USA) supplemented with 15% fetal bovine serum (FBS; Sigma, St. Louis, MO, USA), 1% penicillin-streptomycin (Gibco, Grand Island, NY, USA), and 1% Plasmocin^®^ treatment reagent (InvivoGen, San Diego, CA, USA) at 37 °C in a humidified atmosphere containing 5% CO_2_. To evaluate the effect of DHM on AGE-induced cellular senescence, cells were treated with 200 μg/mL AGEs (Bioss, Beijing, China) in the presence or absence of DHM for either 24 or 48 h. DMSO was used as the solvent for DHM at a final concentration of 0.025%.

### 2.6. Cell Viability Assay

Cells were seeded in 96-well plates at a density of 2000 cells per well and treated with varying concentrations of AGEs and/or DHM. The plates were incubated at 37 °C in a humidified atmosphere with 5% CO_2_. Cell viability was assessed using a CCK-8 assay kit (Beijing LABLEAD Trading Co., Ltd., Beijing, China) according to the manufacturer’s protocol.

### 2.7. Senescence-Associated β-Galactosidase (SA-β-Gal) Staining

SA-β-Gal activity, a widely recognized biomarker of cellular senescence, was detected using the Cell Senescence β-Galactosidase Staining Kit (Vazyme Biotech, Nanjing, China), following the manufacturer’s instructions. The total number of cells and SA-β-Gal-positive cells were counted, and the percentage of positive cells was calculated.

### 2.8. Immunofluorescence Staining

Immunofluorescence staining was used to detect H3K9me2/3, γH2AX, and Ki67 in cells, serving as markers of senescence-associated heterochromatin foci (SAHF), DNA damage, and cell proliferation, respectively [32,33]. Cells were fixed with 4% PFA for 15 min and then blocked for 1 h at room temperature using a blocking buffer containing 1× phosphate-buffered saline (PBS), 5% normal goat serum (Boster Biological Technology, Wuhan, China), and 0.3% Triton X-100 (Solarbio Lifesciences, Beijing, China). Cells were incubated overnight at 4 °C with primary antibodies against H3K9me2/3 (1:100, #5327, Cell Signaling Technology, Danvers, MA, USA), γH2AX (1:200, #9718, Cell Signaling Technology, Danvers, MA, USA), or Ki67. After washing with PBS, the cells were incubated for 1–2 h at room temperature in the dark with an Alexa Fluor 488-conjugated secondary antibody (1:1000, #4412, Cell Signaling Technology, Danvers, MA, USA). Following PBS washes, the nuclei were counterstained with DAPI (#S2110, Solarbio Lifesciences, Beijing, China). Images were acquired using an FV3000 confocal microscope (Olympus, Tokyo, Japan) and analyzed using the Fiji2 (version 2.9.0) software. The fields were randomly selected based on DAPI-stained nuclei.

### 2.9. Western Blot

A total of 250 μL of pre-cooled PBS containing 1% protease inhibitor and 1% phosphatase inhibitor was added to T25 culture flasks. Cells were lysed by ultrasonication (90 W, 4 cycles with 30-s intervals), and the samples were kept on ice throughout the process. Lysates were centrifuged at 12,000 rpm for 5 min at 4 °C, and the supernatants were carefully collected. Protein concentrations were measured using a BCA protein assay kit (Beijing LABLEAD Trading Co., Ltd., Beijing, China). After electrophoresis, the proteins were transferred onto PVDF membranes using a transfer apparatus (400 mA, 40 min). The PVDF membranes were then blocked for 1 h at room temperature with 5% (*w*/*v*) non-fat dry milk in Tris-buffered saline containing 0.1% Tween-20 (TBST). Then, the blocked PVDF membranes were incubated overnight at 4 °C with rabbit monoclonal antibodies against p21^Waf1/Cip1^ (1:1000, #2947, Cell Signaling Technology, Danvers, MA, USA) or β-actin (1:1000, #4970, Cell Signaling Technology, Danvers, MA, USA). After washing with TBST, the membranes were incubated with an HRP-conjugated anti-rabbit secondary antibody (1:3000, #7074, Cell Signaling Technology, Danvers, MA, USA) for 1 h at room temperature. Finally, protein bands were visualized using a chemiluminescence imaging analysis system (UVITEC Ltd., Cambridge, UK), and band intensities were quantified using the Fiji2 software.

### 2.10. Measurement of MMPs in Cultured Cells

Proteins were extracted using ultrasonication, and the entire process was performed on ice. After extraction, the samples were centrifuged at 2000–3000 rpm for 20 min at 4 °C, and the supernatants were carefully collected. The levels of MMP-1, MMP-3, and COL1A1 were quantified using ELISA kits (Nanjing Jiancheng Bioengineering Institute, Nanjing, China) according to the manufacturer’s instructions.

### 2.11. Transcriptome Sequencing and Analysis

Total RNA was extracted using the TRIzol reagent (invitrogen, Carlsbad, CA, USA). RNA concentration and purity were measured using a NanoDrop 2000 spectrophotometer (Thermo Fisher Scientific, Wilmington, DE, USA), and RNA integrity was assessed using an Agilent Bioanalyzer 2100 system (Agilent Technologies Inc., Santa Clara, CA, USA). For library preparation, 1 μg of total RNA per sample was used to generate sequencing libraries using the Hieff NGS Ultima Dual-mode mRNA Library Prep Kit (Yeasen Biotechnology Shanghai Co., Ltd., Shanghai, China), incorporating index codes for sample identification. mRNA was isolated using poly T oligo-attached magnetic beads. cDNA synthesis was performed in two steps, followed by enzymatic modification to produce blunt-ended DNA fragments. Adapter ligation and purification were then performed, followed by PCR amplification. The final quality was assessed using an Agilent Bioanalyzer 2100. Sequencing was performed using the Illumina NovaSeq 6000 platform (Illumina, Inc., San Diego, CA, USA) in PE150 mode. To obtain clean reads, raw reads were filtered using fastp (version 0.22) to remove adapter sequences, poly N reads, and low-quality reads. Clean reads were aligned to the human reference genome (GRCh38) using HISAT2 (version 2.0.4), and differential gene expression analysis was performed using DESeq2 based on a negative binomial distribution model. The *p*-values were adjusted using the false discovery rate (FDR) with the Benjamini-Hochberg method. Genes with adjusted *p*-values < 0.05 and fold change (FC) ≥ 1.5 were identified as differentially expressed genes (DEGs). Gene Set Enrichment Analysis (GSEA) was conducted using the clusterProfiler package in R (version 4.4), with reference gene sets obtained from MSigDB.

### 2.12. Quantitative Real-Time PCR (qRT-PCR)

Total RNA was extracted, and reverse transcription was performed using the GoScript^TM^ Reverse Transcription Mix Kit (Promega, Madison, WI, USA), according to the manufacturer’s protocol. Quantitative real-time PCR (qRT-PCR) was performed using the NovoStart^®^ SYBR qPCR SuperMix Plus kit (Novoprotein Scientific Inc., Suzhou, China) on a CFX96 detector (Bio-Rad, Hercules, CA, USA). Primer sequences are listed in Table 1. The relative expression levels of the target genes were calculated using the 2^−∆∆Ct^ method [34]. *GAPDH* was used as the reference gene for normalization in the cell experiments.

### 2.13. Measurement of Reactive Oxygen Species

Reactive oxygen species (ROS) levels were assessed using a Reactive Oxygen Species Assay Kit (Beyotime Biotechnology, Shanghai, China) according to the manufacturer’s instructions. Fluorescence intensity was measured using a BioTek Synergy Neo2 instrument (Agilent Technologies Inc., Santa Clara, CA, USA) with excitation at 488 nm and emission at 525 nm.

### 2.14. RAGE and Inflammatory Chemokines Analysis

Skin tissue was rapidly frozen in liquid nitrogen and homogenized using a tissue grinder. Subsequently, 0.1 g of tissue was weighed and mixed with 0.9 mL of tissue lysis buffer (Beyotime Biotechnology, Shanghai, China) supplemented with 1% protease inhibitors and 1% phosphatase inhibitors. Samples were lysed on ice using an ultrasonic homogenizer for three cycles and centrifuged at 12,000 rpm for 5 min at 4 °C to collect the supernatant. The levels of RAGE, tumor necrosis factor-alpha (TNF-α), interleukin-1β (IL-1β), and interleukin-6 (IL-6) in the skin tissue were quantified using ELISA kits (all from Boster Biological Technology, China), following the manufacturer’s instructions.

### 2.15. Molecular Docking

Molecular docking was performed to investigate the interaction between DHM and RAGE. The molecular structure of DHM was downloaded from the PubChem database (https://pubchem.ncbi.nlm.nih.gov/, CID:161557), and the molecular structure of the RAGE was obtained from the RCSB Protein Data Bank (https://www.rcsb.org/, PDB ID: 3O3U). FPS-ZM1, a known RAGE receptor inhibitor, was used as a positive control. The structure was obtained from the PubChem database (CID:24752728). Docking simulations were performed using AutoDock v4.2 software, where lower binding energy values indicated a stronger binding affinity and greater conformational stability. A binding energy of less than −5 kcal/mol was considered indicative of a favorable interaction. Docking results were visualized using PyMOL (version 2.5.4).

### 2.16. Surface Plasmon Resonance (SPR)

The binding kinetics and affinity of DHM for RAGE were evaluated using a Biacore 1S+ SPR system (Cytiva, Marlborough, MA, USA) equipped with a CM5 chip. DHM has a molecular weight of 320.25 g/mol, while the RAGE protein (Novoprotein Scientific Inc., Suzhou, China) has a molecular weight of 35.2 kDa, comprising the amino acid sequence from alanine at position 23 to alanine at position 344. The isoelectric point of RAGE was 5.83.

A pre-concentration experiment identified pH 5.5 as the optimal ligand-immobilization condition. Ligand coupling was then performed under the following conditions: coupling amount of approximately 10,000 RU, contact time of 420 s, flow rate of 10 μL/min, and experimental temperature maintained at 25 °C. After immobilization, the interaction between DHM/FPS-ZM1 and RAGE was analyzed using a multi-cycle kinetics method with a contact time of 60 s, flow rate of 20 μL/min, and dissociation time of 300 s. The data were analyzed using Biacore Evaluation Software (1S+ version 1.0).

### 2.17. Statistical Analysis

Data were analyzed using SPSS 27 and OriginPro 2024b and are presented as mean ± SEM. Normality was tested using the Kolmogorov–Smirnov test. For normally distributed data, a one-way ANOVA was performed, followed by pairwise comparisons using the Tukey-Kramer post hoc test. The Kruskal−Wallis test was applied to data that did not satisfy the normal distribution. Statistical significance was set at *p* < 0.05.

## 3. Results

### 3.1. DHM Attenuated Skin Aging in Glycated Rats

**Figure 1:**
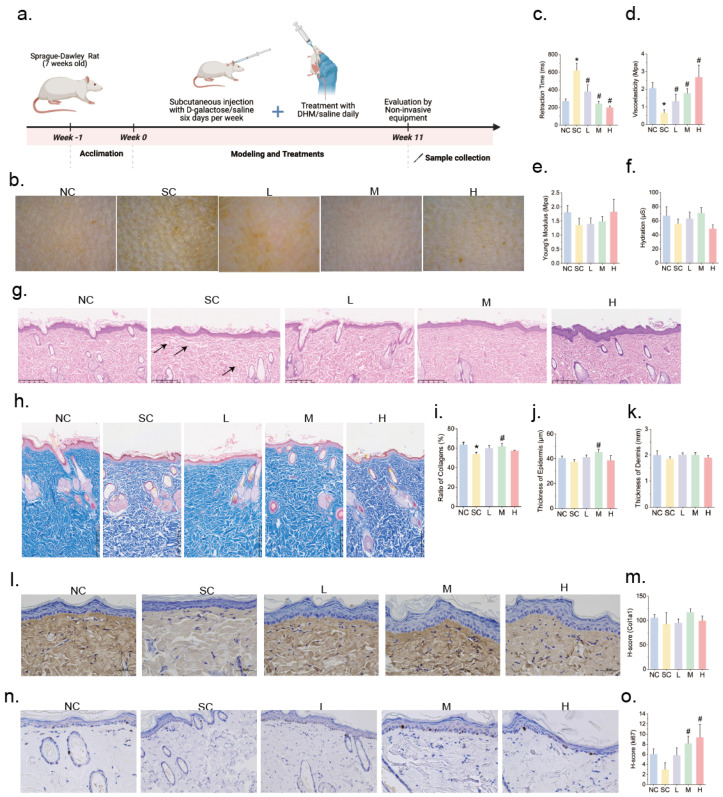
DHM attenuates skin aging in rats. (**a**) Schematic diagram of the treatment strategy in rats; (**b**) Representative dermascopic images (50×) of dorsal skin; Measurement of (**c**) retraction time, (**d**) viscoelasticity, and (**e**) Young’s modulus to evaluate skin elasticity by the elasticity probe per group (*n* = 8); (**f**) Measurement of skin hydration using a hydration pin probe (*n* = 8); (**g**) Representative hematoxylin and eosin (H&E) staining of skin tissue (*n* = 8) with the black arrowheads indicating obviously loose and disorganized sites (scale = 250 μm); (**h**) Representative images of collagen fibers stained by Masson’s trichrome staining (scale = 250 μm) and (**i**) quantification of collagen ratio (*n* = 8); Thickness measurement of (**j**) epidermis and (**k**) dermis based on histological staining per group (*n* = 8); (**l**) Representative images of Col1a1 expression detected by immunohistochemistry (IHC), in which positive staining was shown in brown, scale = 50 μm; (**m**) Quantification of Col1a1 expression using H-score per group (*n* = 3); (**n**) Representative IHC images of Ki67 (400×), in which positive nuclei were stained brown; (**o**) Quantification of Ki67 using H-score per group (*n* = 3). Data are presented as mean ± SEM. * *p* < 0.05 vs. NC; # *p* < 0.05 vs. SC. Abbreviations for treatment groups: NC, normal control; SC, aging group treated with a solvent solution; L, low-dose DHM; M, moderate-dose DHM; H, high-dose DHM.

To evaluate the potential of DHM in mitigating glycation-induced skin aging in rats, eight-week-old rats were subcutaneously injected with D-galactose and simultaneously administered varying doses of DHM via gavage for 11 weeks (Figure 1a)**.** At the end of the experimental period, a handheld digital skin microscope was used to assess the skin texture and wrinkles in the dorsal region. Compared with the NC group, the skin of rats in the SC group appeared visibly rougher, with fine wrinkles and pronounced surface textures characteristic of intrinsic aging. In contrast, the DHM-treated groups (low, moderate, and high doses) showed varying degrees of improvement in skin texture and wrinkle reduction (Figure 1b). Further analysis using the elasticity probe of the DermaLab^®^ Combo3 revealed a significant reduction in skin elasticity in the SC group compared with the NC group, as evidenced by prolonged skin retraction time and decreased viscoelasticity. However, DHM treatment significantly shortened the retraction time and increased viscoelasticity, indicating enhanced skin elasticity (Figure 1c–e). No significant differences were observed in skin hydration between the groups (Figure 1f). Serum levels of AGEs were significantly elevated in the D-galactose-treated groups, indicating the successful induction of glycation. However, DHM treatment did not significantly reduce serum AGE levels (Figure S1).

Skin samples were collected for histopathological analyses. H&E staining revealed disorganized and loose dermal fibers in the SC group, while this condition was alleviated in the DHM-treated groups, particularly in the M and H groups (Figure 1g). Masson’s trichrome staining revealed a significantly lower collagen content in the SC group compared to the NC group. Notably, moderate-dose DHM treatment significantly increased collagen content (Figure 1h,i). While no significant differences in overall epidermal and dermal thickness were observed among the groups, the M group exhibited significantly increased epidermal thickness compared to the SC group (Figure 1j,k). Type I collagen, the most abundant collagen in the skin, was further evaluated using immunohistochemistry (IHC). No significant differences in Col1a1 levels were observed among the groups (Figure 1l,m). However, a decreasing trend in Ki67 levels was noted in the aging group, whereas DHM treatment resulted in a dose-dependent increase in Ki67 expression (Figure 1n,o), suggesting that DHM promoted skin cell proliferation and helped maintain normal skin structure.

In summary, DHM effectively attenuated glycation-induced skin aging in rats by enhancing skin elasticity, promoting skin cell proliferation, and preserving collagen levels.

### 3.2. DHM Delayed AGE-Induced Cellular Senescence

**Figure 2:**
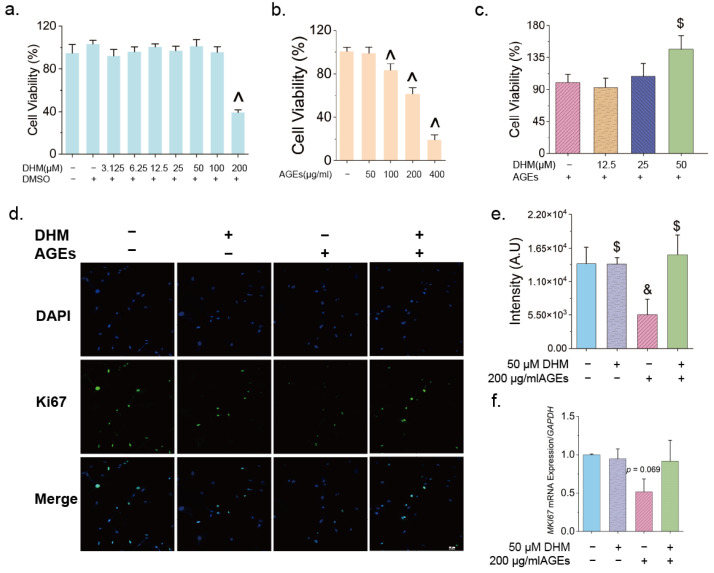
DHM promoted cell proliferation impaired by AGEs. Cell viability was assessed using the CCK-8 assay following treatment with (**a**) DHM and (**b**) AGEs (*n* = 6); (**c**) Evaluation of cell viability after five days of co-treatment with 50 μM DHM and 200 μg/mL AGEs (*n* = 5); (**d**) Representative confocal images showing Ki67staining (scale = 40 μm) and (**e**) Quantification of fluorescence intensity of Ki67 (*n* = 3); (**f**) mRNA expression of *MKI67* in HFF-1 cells treated with 50 μM DHM and/or 200 μg/mL AGEs (*n* = 3). Data are presented as mean ± SEM. ^ *p* < 0.05 vs. untreated control; $ *p* < 0.05 vs. AGEs; & *p* < 0.05 vs. cells cultured in complete medium containing 0.025% DMSO content.

As shown above, DHM enhanced cell proliferation in rat skin, suggesting its potential role in mitigating skin aging through this mechanism. To further investigate whether DHM alleviates the effects of AGEs on skin cell proliferation and delays AGE-induced cellular senescence, cell viability assays were conducted. First, the individual effects of AGEs and DHM on cell growth were assessed. The results showed that 100 μg/mL AGEs significantly reduced cell viability, whereas DHM at concentrations below 100 μM had a relatively minimal impact (Figure 2a,b). Treatment with 50 μM DHM markedly improved cell viability in AGE-exposed cells, increasing survival rates by 1.47 ± 0.28-fold and 1.58 ± 0.38-fold on days 5 and 7, respectively (Figure 2c and Figure S2). AGEs significantly suppressed both mRNA and protein expression of Ki67, a proliferation biomarker, while 50 μM DHM substantially restored Ki67 levels (Figure 2d–f). These findings further confirm that DHM effectively counteracts AGE-induced decline in cell proliferation.

**Figure 3:**
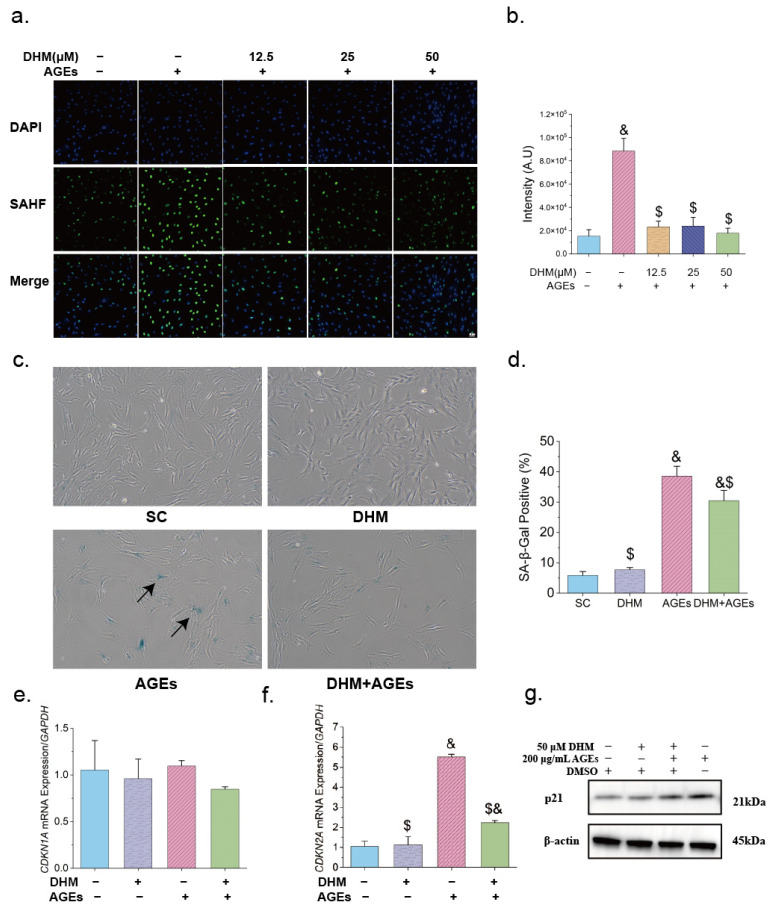
DHM delayed cellular senescence induced by AGEs in HFF-1 cells. (**a**) Representative confocal images of SAHF treated with various concentrations of DHM and 200 μg/mL AGEs, scale = 40 μm; (**b**) Quantification of fluorescence intensity of SAHF (*n* = 4); (**c**) Representative image of senescence-associated β-galactosidase (SA-β-Gal) staining in cells (100×) treated with 50 μM DHM and/or 200 μg/mL AGEs, with blue-stained cells (indicated by dark arrows) representing SA-β-Gal-positive cells; (**d**) Quantification of the percentage of SA-β-Gal-positive cells (*n* = 6); mRNA expression levels of (**e**) *CDKN1A* and (**f**) *CDKN2A* following treatment with 50 μM DHM and 200 μg/mL AGEs (*n* = 3); (**g**) Representative Western blot showing p21 expression in treated cells. Data are presented as mean ± SEM. $ *p* < 0.05 vs. AGEs; & *p* < 0.05 vs. SC. Abbreviation for groups: SC, cells cultured with complete medium containing 0.025% DMSO; AGEs group, cells treated with 200 μg/mL AGEs; DHM, cells treated with 50 μM DHM; DHM + AGEs, cells treated with 50 μM DHM and 200 μg/mL AGEs.

As cell proliferation level is a key indicator of cellular senescence, we next assessed several classical senescence-associated markers. AGEs significantly increased the formation of SAHF in the nuclei (Figure 3a,b) and elevated the proportion of SA-β-Gal-positive cells (Figure 3c,d). In addition, AGEs upregulated the mRNA expression of p16 (encoded by *CDKN2A*) and protein expression of p21 (Figure 3e–g). However, DHM significantly delayed AGE-induced cellular senescence. At concentrations ranging from 12.5 to 50 μM, DHM markedly suppressed SAHF formation, with more pronounced effects observed at higher doses (Figure 3a,b). Furthermore, 50 μM DHM significantly reduced the proportion of SA-β-Gal-positive cells and downregulated the expression of cyclin-dependent kinase inhibitors (CDKIs), as shown in Figure 3c–g.

In summary, DHM effectively promoted cell proliferation, alleviated AGE-induced reductions in cell viability, and delayed cellular senescence induced by AGEs.

### 3.3. Effects of DHM on MMPs and COL1A1 Expression Induced by AGEs in HFF-1 Cells

**Figure 4:**
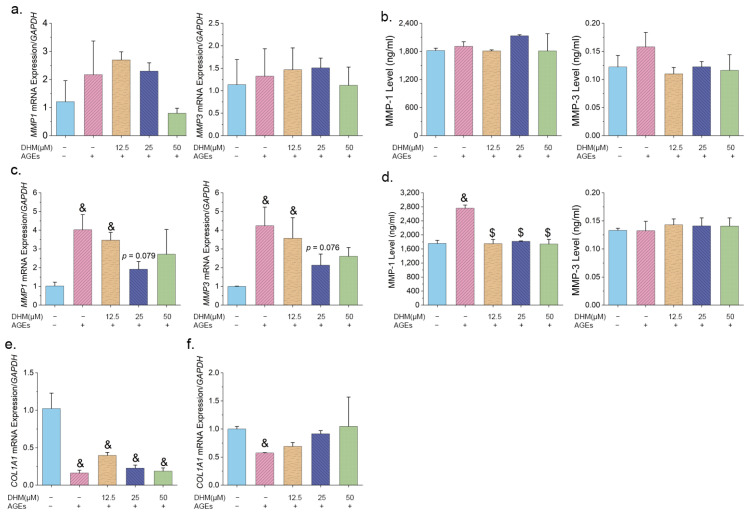
DHM inhibited MMP1 secretion induced by AGEs in HFF-1 cells. (**a**) mRNA expression levels of *MMP1* and *MMP3* and (**b**) corresponding protein levels in cells treated with various concentrations of DHM and 200 μg/mL AGEs for 24 h; (**c**) mRNA expression levels of *MMP1* and *MMP3* and (**d**) corresponding protein levels after treatment for 48 h; mRNA expression of *COL1A1* following treatment for (**e**) 24 h and (**f**) 48 h. Data are presented as mean ± SEM. $ *p* < 0.05 vs. AGEs; & *p* < 0.05 vs. cells cultured in complete medium containing 0.025% DMSO.

MMPs are critical markers of skin aging and are closely associated with collagen degradation. After 24 h of treatment, AGEs did not significantly alter *MMP1* or *MMP3* expression in HFF-1 cells (Figure 4a,b). However, after 48 h, *MMP1* and *MMP3* gene expression levels were significantly upregulated, and this effect was partly reversed by 25 μM DHM treatment (Figure 4c). Compared to senescent cells induced by 200 μg/mL AGEs, DHM treatment at concentrations of 12.5 μM, 25 μM, and 50 μM markedly reduced MMP-1 secretion (Figure 4d). In contrast, no significant differences were observed in MMP-3 protein levels among the groups. In addition to collagen degradation, impaired collagen synthesis contributes to skin aging. AGEs severely downregulated *COL1A1* mRNA expression at both 24 h and 48 h. However, treatment with DHM for 48 h partially restored the *COL1A1* expression (Figure 4e,f). No significant differences in COL1A1 protein levels were observed among the groups (Table S1).

In summary, AGEs induced MMP-1 expression and promoted collagen degradation, while DHM effectively inhibited MMP-1 secretion and showed potential for mitigating collagen breakdown.

### 3.4. DHM Might Mtigate Cellular Senescence by Targeting AGE-RAGE Signaling

To investigate the mechanisms by which DHM alleviates AGE-induced cellular senescence and enhances cell proliferation, transcriptomic sequencing was performed to assess the differences in gene expression profiles. As shown in Figure S3a, the expression profiles of AGE-treated cells and DHM-treated cells were clearly distinct. DEG analysis identified a total of 464 DEGs between the AGEs group (treated with 200 μg/mL AGEs) and the DHM+AGE group (treated with 50 μM DHM and 200 μg/mL AGEs), including 206 upregulated and 258 downregulated genes (Figure S3b). A Venn diagram revealed 306 shared DEGs between the comparisons, of which 155 genes were restored in the DHM-treated group (Figure S3c). These genes may serve as key regulators of the anti-senescence effects of DHM.

**Figure 5:**
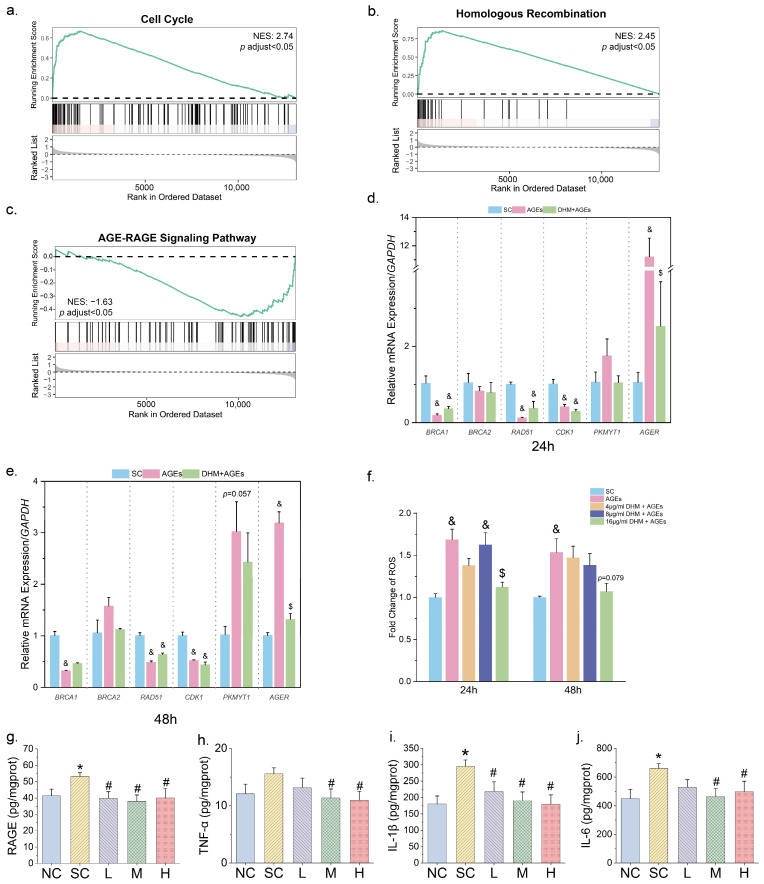
DHM regulated RAGE expression, which was markedly induced by AGEs. Gene Set Enrichment Analysis (GSEA) showing upregulation of (**a**) Cell cycle and (**b**) homologous recombination pathways by 50 μM DHM under 200 μg/mL AGEs stimulation, with positive normalized enrichment score (NES) indicating upregulation; (**c**) Downregulation of AGE-RAGE signaling by DHM (negative NES indicating downregulation); mRNA expression of key Differentially Expressed Genes (DEGs) validated by qRT-PCR in HFF-1 cells treated for (**d**) 24 h and (**e**) 48 h, respectively; (**f**) Measurements of reactive oxygen species (ROS) level; Protein level of (**g**) RAGE, (**h**) TNF-α, (**i**) IL-1β and (**j**) IL-6 in rat skin measured by ELISA per group (*n* = 8). Data are presented as mean ± SEM. $ *p* < 0.05 vs. cells treated with 200 μg/mL AGEs; & *p* < 0.05 vs. cells cultured in complete medium containing 0.025% DMSO; * *p* < 0.05 vs. NC group in rat experiment; # *p* < 0.05 vs. SC group in rat experiment. Abbreviations for treatment groups in (**g**–**j**): NC, normal control; SC, aging group treated with a solvent solution; L, low-dose DHM; M, moderate-dose DHM; H, high-dose DHM.

GSEA revealed that, compared with non-senescent cells, the AGEs group exhibited significant upregulation of pathways related to proteasome activity, FcεRI-mediated NF-κB activation, ROS signaling, type I diabetes, TP53-regulated death receptor-ligand transcription, and aging (Figure S4a). In contrast, the pathways involved in the cell cycle, DNA replication, homologous recombination (HR), mismatch repair, and chromosome maintenance were downregulated in the AGEs group (Figure S4a). Compared with the AGEs group, the DHM + AGEs group exhibited upregulation of DNA replication, cell cycle, and aging-related TP53 target downregulated gene sets, HR, and mismatch repair pathways. Simultaneously, there was a marked downregulation of inflammation- and aging-related pathways, such as the AGE-RAGE signaling pathway, Notch1 signaling pathway, IL-8/CXCR2 and IL-8/CXCR1 pathways, GPCR-PI3K signaling, NF-κB signaling, and TNF signaling (Figure 5a–c and Figure S4b). These findings suggest that DHM restores the expression of genes involved in cell cycle regulation, DNA repair, HR, and mismatch repair while concurrently suppressing inflammation- and aging-related signaling pathways.

Further analysis of key DEGs in the major enriched pathways identified several genes with high FC, including *BRCA1*, *BRCA2*, *RAD51*, *CDK1*, *PKMYT1*, and *AGER*, which were involved in multiple signaling pathways. qRT-PCR validation confirmed that 200 μg/mL AGEs significantly downregulated the expression of *BRCA1* and *RAD51* (two critical genes in HR), as well as cell cycle-dependent kinase CDK1. Moreover, AGEs tended to upregulate the mRNA expression of PKMYT1 (a CDK1 kinase) with an extended treatment duration. AGEs also significantly upregulated the gene expression of RAGE (Figure 5d,e). Notably, 50 μM DHM treatment effectively downregulated *AGER* (the gene encoding RAGE), highlighting its potential role in inhibiting AGE-RAGE signaling activation. Additionally, GSEA analysis indicated increased ROS signaling following AGE treatment. As shown in Figure 5f, AGEs significantly elevated intracellular ROS production, while 50 μM DHM markedly reduced ROS accumulation in cells.

Prompted by these findings, we further investigated the expression of RAGE and related molecules in rat skin to evaluate the in vivo relevance of DHM treatment. DHM administration, particularly at moderate and high doses, attenuated the glycation-induced upregulation of RAGE expression in the skin of rats (Figure 5g). Pro-inflammatory chemokines downstream of RAGE, including IL-6 and IL-1β, were elevated in glycated rats, indicating the activation of AGE–RAGE signaling. Importantly, DHM treatment significantly reduced the secretion of several key chemokines, including TNF-α, IL-6, and IL-1β (Figure 5h–j), further supporting the role of DHM in regulating RAGE-mediated inflammatory responses.

### 3.5. DHM May Function as an Inhibitor of RAGE Against Aging

Previous studies have reported that DHM inhibits multiple downstream pathways of RAGE. Based on the findings presented above, the role of DHM in the regulation of *AGER* expression may exert a wide inhibitory effect on RAGE signaling. Supporting this hypothesis, several RAGE-associated pathways, including the GPCR-PI3K and NF-κB signaling pathways, were downregulated by DHM (Figure S4b). These results imply that DHM may function as an upstream regulator of RAGE.

**Figure 6:**
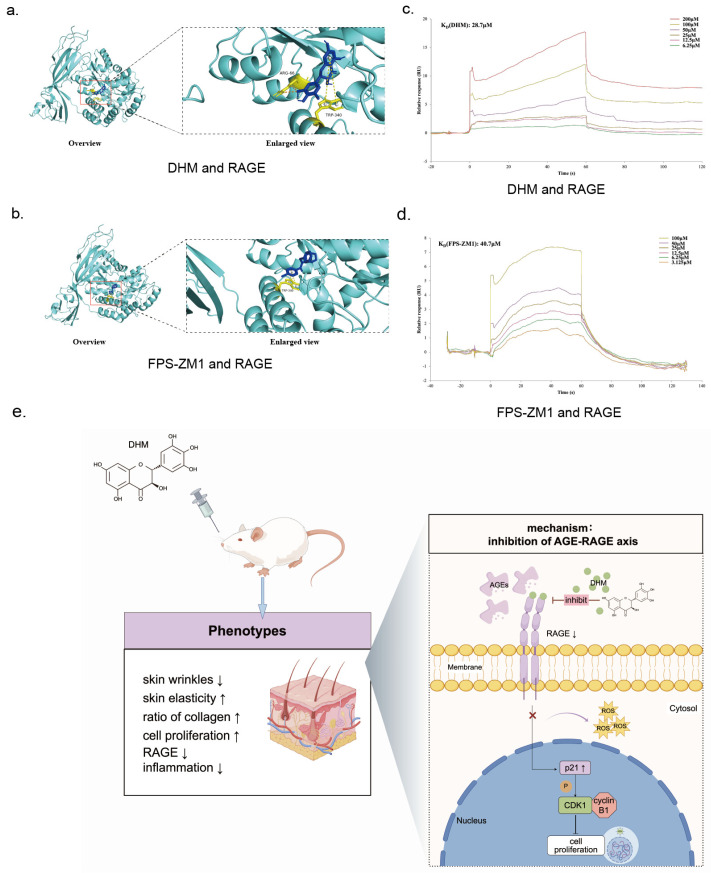
DHM may function as an RAGE inhibitor to mitigate skin aging. (**a**) Molecular docking analysis showing the interaction site between DHM and RAGE; (**b**) molecular docking analysis of the interaction between FPS-ZM1 and RAGE; (**c**) surface plasmon resonance (SPR) binding curve of DHM and RAGE; (**d**) SPR binding curve of FPS-ZM1 and RAGE; (**e**) central illustration of this study, drawn by Figdraw (ID: RWSWP8328b).

To verify this hypothesis, molecular docking analyses were performed to evaluate the interaction between DHM and RAGE, using the known RAGE inhibitor FPS-ZM1 as a positive control. The docking results showed that both DHM and FPS-ZM1 are stably bound to the same region of the RAGE protein (Figure 6a,b). The minimum binding energy of DHM to RAGE was −9.4 kcal/mol, involving interactions with residues Lys15, Trp62, Ala63, Glu111, Glu153, Pro154, Tyr155, Phe156, and Trp340. DHM formed two hydrogen bonds with Arg66 and two T-shaped π-π stacking interactions with Trp340 (Figure 6a). In comparison, FPS-ZM1 exhibited minimum binding energy of −9.1 kcal/mol and also formed two T-shaped π-π stacking interactions at Trp340 (Figure 6b). Based on the binding conformations and energies, DHM exhibited slightly stronger binding stability with RAGE than FPS-ZM1.

To further validate this interaction, we performed an SPR analysis. Both DHM and FPS-ZM1 were bound to RAGE in a dose-dependent manner. DHM exhibited a dissociation constant (K_D_) of 28.7 μM, while FPS-ZM1 showed a K_D_ of 40.7 μM, indicating a higher binding affinity of DHM. Moreover, FPS-ZM1 demonstrated a faster dissociation rate upon injection cessation, whereas DHM maintained its binding state for a longer duration, suggesting a more stable interaction with RAGE (Figure 6c,d).

In conclusion, the findings of the current study suggest that DHM functions as a RAGE inhibitor, thereby suppressing downstream signaling activation, reducing pro-inflammatory chemokine release, and ultimately alleviating skin aging.

## 4. Discussion

In recent years, endogenous factors contributing to skin aging have garnered increasing attention. Unlike photoaging, endogenous aging has a more profound impact on overall skin health. This study focused on glycation-induced skin aging and demonstrated that DHM significantly alleviated this condition in rats. Furthermore, we identified DHM as a potential RAGE inhibitor, suggesting its role in suppressing AGE-RAGE pathway activation, delaying cellular senescence, and promoting skin cell proliferation.

Previous studies have primarily investigated the role of DHM in UV-induced skin aging. In this study, we report for the first time that DHM also exerts anti-aging effects on glycation-induced skin aging using a rat model induced by D-galactose. This model has been widely used in studies associated with aging, including skin aging studies [31,35]. The current study demonstrated that DHM treatment increased dermal collagen content and concurrently reduced skin inflammation in rats. These findings were similar to those of previous observations in UV-induced models; however, histological changes indicating skin aging were milder than those in photoaging, as previously reported. In a murine model of skin photoaging [29], DHM significantly alleviated erythema, wrinkle formation, and fluid exudation. DHM reduced the levels of TNF-α, IL-1β, mast cells, and MMP-1, thereby mitigating inflammation and collagen degradation. Another study demonstrated that topical application of DHM to UV-exposed skin significantly promoted skin regeneration, as evidenced by increased epidermal thickness and higher dermal cell counts [27]. Similarly, our study also revealed that DHM enhanced overall cell proliferation in aged skin, providing compelling evidence for its role in ameliorating endogenous skin aging.

Cellular senescence is a principal hallmark of aging and a key contributor to aging-related diseases [32,36,37], including skin aging [38]. This study focused on the detrimental effects of AGEs and demonstrated that AGEs significantly accelerated cellular senescence, a phenomenon confirmed across multiple cell types [39,40]. Notably, AGE-induced senescence was markedly delayed in HFF-1 cells treated with DHM, which exhibited proliferative effects similar to those observed in UV-induced aging models [28,29]. These findings underscore the potent anti-senescence and pro-proliferative properties of DHM. Transcriptomic analysis revealed the underlying mechanisms by which AGEs accelerated senescence and how DHM mitigated these effects. AGEs markedly disrupted pathways involved in HR and cell cycle progression, particularly by downregulating the mRNA expression of *BRCA1*, *RAD51*, and *CDK1*. BRCA1, a tumor suppressor encoded by *BRCA1*, plays a critical role in repairing DNA double-strand breaks (DSB) via HR [41]. BRCA1 interacts with PALB2 to recruit BRCA2 to DSB sites, facilitating the loading of RAD51, a core HR repair enzyme. RAD51 then promotes strand invasion and displacement loop (D-loop) formation, utilizing sister chromatids as templates to synthesize DNA across DSB sites, thereby preserving genomic integrity [42]. Our study demonstrated that AGEs severely impair HR repair. Cyclin-dependent kinase 1 (CDK1), the first identified member of the CDK family, is a highly conserved protein that plays a key role in regulating the G2-to-M phase transition of the cell cycle [43]. CDK1 forms a complex with cyclin B1, known as the pre-mitosis Promoting Factor (pre-MPF). When the Tyr15 and Thr14 residues of CDK1 are phosphorylated by kinases such as PKMYT1, the complex becomes inactive, resulting in cell cycle arrest at the G2/M phase. Conversely, dephosphorylation of these residues activates pre-MPF, enabling cells to enter mitosis. In our study, prolonged incubation increased PKMYT1 expression in senescent cells. These findings suggest that AGEs not only downregulate CDK1 gene expression but also upregulate kinases that promote CDK1 phosphorylation, thereby impairing cell proliferation. Interestingly, CDK1 expression did not significantly increase in the DHM treatment group. This observation implies that the proliferative effects of DHM may not be due to direct enhancement of CDK1 expression but rather through the downregulation of CDKIs (e.g., p21), leading to reduced CDK1 phosphorylation.

Notably, AGE-RAGE signaling was significantly downregulated following DHM treatment, particularly through the suppression of RAGE expression. As the primary receptor for AGEs, RAGE is widely expressed in the skin and is recognized as a key therapeutic target for dermal diseases [44]. AGEs can upregulate the expression of RAGE, activating the RAGE-NF-κB signaling pathway and leading to the formation of a positive feedback loop between NF-κB and RAGE [45]. This mechanism contributes to inflammatory senescence, characterized by elevated RAGE expression and increased secretion of pro-inflammatory chemokines, including IL-1β, IL-6, and TNF-α [46]. In addition to its role in senescence, numerous studies using RAGE-knockout animal models have demonstrated a correlation between RAGE and cell proliferation [47]. Increased RAGE expression has been shown to significantly inhibit cell proliferation and induce G1 phase cell cycle arrest, accompanied by upregulation of senescence-associated markers, such as p53 and p21 [48]. In our study, DHM significantly reduced the expression of *AGER*. Based on these findings, we hypothesized that the pro-proliferative effect of DHM might be mediated by inhibiting AGE-RAGE signaling. Moreover, activation of AGE-RAGE signaling stimulates ROS production [49,50,51], which exacerbates DNA damage and promotes cellular senescence [52,53]. DHM exhibits potent ROS-scavenging activity [54]. Consistent with this, ROS levels were markedly reduced in DHM-treated cells in our study (Figure 5f). As a result, DNA damage was alleviated, the expression of senescence-associated molecules was downregulated, and cellular senescence was delayed.

The correlation between DHM and AGE-RAGE signaling has been reported in patients with diabetes and depression; however, these findings were primarily derived from network pharmacology analyses [55,56]. A recent transcriptomic study demonstrated that Zn-DHM nanozymes (a novel metal-polyphenolic nanozyme synthesized by the coordination of Zn^2+^ with DHM) could downregulate the AGE-RAGE signaling pathway [54], which is consistent with our findings. Additionally, the in vivo study provided further evidence supporting the inhibitory effect of DHM on the AGE-RAGE signaling pathway. Among the downstream pathways regulated by RAGE [44,57], DHM has been reported to interact with multiple pathways, including PI3K-Akt, MAPK/NF-κB, and c-Jun *N*-terminal kinase (JNK) [58]. These findings imply the potential role of DHM as an upstream regulator of the AGE-RAGE axis, exerting broad inhibitory effects on RAGE-related downstream pathways. Nevertheless, a direct interaction between DHM and RAGE has not been previously reported. To explore this possibility, we conducted molecular docking and SPR analyses and confirmed our hypothesis. We found that DHM bound to the same domain on RAGE as the known inhibitor FPS-ZM1, which corresponds to the ligand-binding domain. We concluded that DHM binding to RAGE disrupted the positive feedback loop and attenuated downstream pathway activation, as observed in rat skin.

The pharmacological activities of DHM have been demonstrated in several systems [59]. Its role in attenuating AGE-induced skin aging has been further confirmed. In addition to studies on its bioactivity, other concerns have attracted attention. Low bioavailability is a common limitation of many phytochemicals, including DHM. However, the distribution of DHM has been observed in various tissues, including the plasma, intestine, brain, and other organs [60]. Further data are needed regarding its distribution in the skin. Although DHM is present at relatively low concentrations in vivo, its cumulative effects over prolonged treatment durations may contribute to its therapeutic benefits. Additionally, DHM has been reported to interact with other molecules [27,61]. In this study, we identified a direct interaction between DHM and RAGE. In the future, screening technologies may be employed to further explore potential targets, which may help to elucidate the synergistic effects of DHM. In conclusion, the current study provides the first evidence identifying DHM as a potential RAGE inhibitor, demonstrating a higher binding affinity and a more stable interaction conformation than FPS-ZM1. However, several questions remain to be answered. First, further in vivo studies are needed to further validate the interaction between DHM and RAGE. Second, the specific downstream pathways activated or inhibited by DHM binding to RAGE require further investigation. These gaps will be addressed in future research.

## 5. Conclusions

This study elucidates the role of DHM in attenuating glycation-induced skin aging in rats. Furthermore, our findings indicate that DHM may serve as a potential RAGE inhibitor, suppressing AGE-RAGE signaling activation and promoting skin cell viability.

## Figures and Tables

**Table 1:** Primer sequences for real-time PCR.

Gene	Forward Primer (5′ to 3′)	Reverse Primer (5′ to 3′ )
*CDKN1A*	TGTCCGTCAGAACCCATGC	AAAGTCGAAGTTCCATCGCTC
*CDKN2A*	GGGTTTTCGTGGTTCACATCC	CTAGACGCTGGCTCCTCAGTA
*MKI67*	ACGCCTGGTTACTATCAAAAGG	CAGACCCATTTACTTGTGTTGGA
*MMP1*	AAAATTACACGCCAGATTTGCC	GGTGTGACATTACTCCAGAGTTG
*MMP3*	AAGGATACAACAGGGACCA	GTTGGCTGAGTGAAAGAGAC
*COL1A1*	GTGCGATGACGTGATCTGTGA	CGGTGGTTTCTTGGTCGGT
*BRCA1*	TTGTTACAAATCACCCCTCAAGG	CCCTGATACTTTTCTGGATGCC
*BRCA* *2*	TGCCTGAAAACCAGATGACTATC	AGGCCAGCAAACTTCCGTTTA
*RAD51*	CAACCCATTTCACGGTTAGAGC	TTCTTTGGCGCATAGGCAACA
*CDK1*	AAACTACAGGTCAAGTGGTAGCC	TCCTGCATAAGCACATCCTGA
*PKMYT1*	GCCTGCCAACATCTTCCTG	CCCAGACTGAACACATCCGC
*AGER*	CACCTTCTCCTGTAGCTTCAGC	AGGAGCTACTGCTCCACCTTCT
*GAPDH*	ACAGTCAGCCGCATCTTCTT	GTTAAAAGCAGCCCTGGTGA

## Data Availability

Data will be shared upon reasonable request from the corresponding author.

## Supplementary Materials

The following supporting information can be downloaded at: https://www.mdpi.com/article/10.3390/nu17111862/s1, Figure S1: Levels of AGEs in rat serum. *n* = 8; * *p* < 0.05 vs. NC. Figure S2: DHM promoted cell growth influenced by AGEs. Figure S3: PCA analysis and difference in gene count across groups in transcriptomic analysis. Figure S4: The ridgeline plot of Gene Set Enrichment Analysis (GSEA). Table S1: Level of COL1A1 in HFF-1 cells with different treatments (Mean ± SEM, *n* = 3).

## Abbreviations

The following abbreviations are used in this manuscript:

AGEs	advanced glycation end products
BCA	Bicinchoninic Acid Assay
BRCA1	breast cancer type 1 susceptibility protein
BRCA2	breast cancer type 2 susceptibility protein
CDKI	Cyclin-dependent kinase inhibitor
CDKN1A	Cyclin-dependent kinase inhibitor 1A
CDKN2A	Cyclin-dependent kinase inhibitor 2A
COL1A1	collagen type I alpha 1 chain
DAPI	4′,6-Diamidino-2′-phenylindole
DEG	differentially expressed gene
DHM	dihydromyricetin
D-loop	displacement loop
DMEM	Dulbecco’s Modified Eagle Medium
DSB	DNA double-strand break
ECM	extracellular matrix
FBS	fetal bovine serum
FcɛRI	the high-affinity IgE Fc receptor
FC	fold change
FDR	false discovery rate
GAPDH	glyceraldehyde-3-phosphate dehydrogenase
GSEA	Gene Set Enrichment Analysis
H&E	hematoxylin and eosin
HR	homologous recombination
IHC	immunohistochemistry
IL-1β	interleukin-1β
IL-6	interleukin-6
JNK	c-Jun *N*-terminal kinase
K_D_	dissociation constant
KEGG	Kyoto Encyclopedia of Genes and Genomes
MKI67	marker of proliferation Ki-67
MMP	matrix metalloproteinase
MMP-1	matrix metallopeptidase-1
MMP-3	matrix metallopeptidase-3
NF-κB	nuclear factor-κB
PFA	paraformaldehyde
PKMYT1	protein kinase membrane associated tyrosine/threonine 1
pre-MPF	pre-Mitosis Promoting Factor
PVDF	polyvinylidene fluoride
RAD51	RAD51 recombinase
ROS	reactive oxygen species
SAHF	senescence-associated heterochromatin foci
SA-β-Gal	senescence-associated β-galactosidase
SPF	specific pathogen-free
SPR	surface plasmon resonance
TBST	Tris-buffered saline with 0.1% Tween-20
TNF-α	tumor necrosis factor-alpha
UV	ultraviolet

## References

[1] Zouboulis C.C., Ganceviciene R., Liakou A.I., Theodoridis A., Elewa R., Makrantonaki E. (2019). Aesthetic aspects of skin aging, prevention, and local treatment. Clin. Dermatol..

[2] Zhang L.J., Chen S.X., Guerrero-Juarez C.F., Li F., Tong Y., Liang Y., Liggins M., Chen X., Chen H., Li M. (2019). Age-Related Loss of Innate Immune Antimicrobial Function of Dermal Fat Is Mediated by Transforming Growth Factor Beta. Immunity.

[3] Kaur A., Ecker B.L., Douglass S.M., Kugel C.H., Webster M.R., Almeida F.V., Somasundaram R., Hayden J., Ban E., Ahmadzadeh H. (2019). Remodeling of the Collagen Matrix in Aging Skin Promotes Melanoma Metastasis and Affects Immune Cell Motility. Cancer Discov..

[4] Gunn D.A., de Craen A.J.M., Dick J.L., Tomlin C.C., van Heemst D., Catt S.D., Griffiths T., Ogden S., Maier A.B., Murray P.G. (2013). Facial Appearance Reflects Human Familial Longevity and Cardiovascular Disease Risk in Healthy Individuals. J. Gerontol. Ser. A.

[5] Waaijer M.E., Parish W.E., Strongitharm B.H., van Heemst D., Slagboom P.E., de Craen A.J., Sedivy J.M., Westendorp R.G., Gunn D.A., Maier A.B. (2012). The number of p16INK4a positive cells in human skin reflects biological age. Aging Cell.

[6] Slominski A.T., Zmijewski M.A., Plonka P.M., Szaflarski J.P., Paus R. (2018). How UV Light Touches the Brain and Endocrine System Through Skin, and Why. Endocrinology.

[7] Skobowiat C., Slominski A.T. (2015). UVB Activates Hypothalamic-Pituitary-Adrenal Axis in C57BL/6 Mice. J. Investig. Dermatol..

[8] Gu Y., Han J., Jiang C., Zhang Y. (2020). Biomarkers, oxidative stress and autophagy in skin aging. Ageing Res. Rev..

[9] Hussein R.S., Bin Dayel S., Abahussein O., El-Sherbiny A.A. (2025). Influences on Skin and Intrinsic Aging: Biological, Environmental, and Therapeutic Insights. J. Cosmet. Dermatol..

[10] Wong Q.Y.A., Chew F.T. (2021). Defining skin aging and its risk factors: A systematic review and meta-analysis. Sci. Rep..

[11] D’Cunha N.M., Sergi D., Lane M.M., Naumovski N., Gamage E., Rajendran A., Kouvari M., Gauci S., Dissanayka T., Marx W. (2022). The Effects of Dietary Advanced Glycation End-Products on Neurocognitive and Mental Disorders. Nutrients.

[12] Monnier V.M., Sell D.R., Abdul-Karim F.W., Emancipator S.N. (1988). Collagen browning and cross-linking are increased in chronic experimental hyperglycemia. Relevance to diabetes and aging. Diabetes.

[13] Monnier V.M. (1989). Toward a Maillard reaction theory of aging. Prog. Clin. Biol. Res..

[14] Zhu J., Wang Z., Lv C., Li M., Wang K., Chen Z. (2024). Advanced Glycation End Products and Health: A Systematic Review. Ann. Biomed. Eng..

[15] Dozio E., Caldiroli L., Molinari P., Castellano G., Delfrate N.W., Romanelli M.M.C., Vettoretti S. (2023). Accelerated AGEing: The Impact of Advanced Glycation End Products on the Prognosis of Chronic Kidney Disease. Antioxidants.

[16] Rungratanawanich W., Qu Y., Wang X., Essa M.M., Song B.J. (2021). Advanced glycation end products (AGEs) and other adducts in aging-related diseases and alcohol-mediated tissue injury. Exp. Mol. Med..

[17] Singh S., Siva B.V., Ravichandiran V. (2022). Advanced Glycation End Products: Key player of the pathogenesis of atherosclerosis. Glycoconj. J..

[18] Wang L., Jiang Y., Zhao C. (2024). The effects of advanced glycation end-products on skin and potential anti-glycation strategies. Exp. Dermatol..

[19] Chen C.-y., Zhang J.-Q., Li L., Guo M.-m., He Y.-f., Dong Y.-m., Meng H., Yi F. (2022). Advanced Glycation End Products in the Skin: Molecular Mechanisms, Methods of Measurement, and Inhibitory Pathways. Front. Med..

[20] Liu D., Mao Y., Ding L., Zeng X.A. (2019). Dihydromyricetin: A review on identification and quantification methods, biological activities, chemical stability, metabolism and approaches to enhance its bioavailability. Trends Food Sci. Technol..

[21] Zhang X., Zhang L., Zhang Y., Xiong T., Niu Y., Huang Y. (2023). Extracting myricetin and dihydromyricetin simultaneously from Hovenia acerba seed by Ultrasound-Assisted extraction on a lab and small Pilot-Scale. Ultrason. Sonochem..

[22] Liu Y., Li F., Fei T., Lin X., Wang L., Liu Z. (2025). Natural alpha-glucosidase inhibitors from Aquilaria sinensis leaf-tea: Targeted bio-affinity screening, identification, and inhibition mechanism. Food Chem..

[23] Wang Y., Wang J., Xiang H., Ding P., Wu T., Ji G. (2022). Recent update on application of dihydromyricetin in metabolic related diseases. Biomed. Pharmacother..

[24] Fan X., Zeng Y., Fan Z., Cui L., Song W., Wu Q., Gao Y., Yang D., Mao X., Zeng B. (2020). Dihydromyricetin promotes longevity and activates the transcription factors FOXO and AOP in Drosophila. Aging.

[25] Sun C.C., Yin Z.P., Chen J.G., Wang W.J., Zheng G.D., Li J.E., Chen L.L., Zhang Q.F. (2022). Dihydromyricetin Improves Cognitive Impairments in d-Galactose-Induced Aging Mice through Regulating Oxidative Stress and Inhibition of Acetylcholinesterase. Mol. Nutr. Food Res..

[26] Qian J., Wang X., Cao J., Zhang W., Lu C., Chen X. (2021). Dihydromyricetin attenuates D-galactose-induced brain aging of mice via inhibiting oxidative stress and neuroinflammation. Neurosci. Lett..

[27] Falckenhayn C., Bienkowska A., Sohle J., Wegner K., Raddatz G., Kristof B., Kuck D., Siegner R., Kaufmann R., Korn J. (2023). Identification of dihydromyricetin as a natural DNA methylation inhibitor with rejuvenating activity in human skin. Front. Aging.

[28] He Z., Zhang L., Zhuo C., Jin F., Wang Y. (2016). Apoptosis inhibition effect of Dihydromyricetin against UVA-exposed human keratinocyte cell line. J. Photochem. Photobiol. B.

[29] Moon N.R., Kang S., Park S. (2018). Consumption of ellagic acid and dihydromyricetin synergistically protects against UV-B induced photoaging, possibly by activating both TGF-β1 and wnt signaling pathways. J. Photochem. Photobiol. B.

[30] Liu Z., Hu G.D., Luo X.B., Yin B., Shu B., Guan J.Z., Jia C.Y. (2017). Potential of bone marrow mesenchymal stem cells in rejuvenation of the aged skin of rats. Biomed. Rep..

[31] Yuan S., Yang Y., Li J., Tan X., Cao Y., Li S., Hong H.D., Liu L., Zhang Q. (2020). Ganoderma lucidum Rhodiola compound preparation prevent D-galactose-induced immune impairment and oxidative stress in aging rat model. Sci. Rep..

[32] Lopez-Otin C., Blasco M.A., Partridge L., Serrano M., Kroemer G. (2023). Hallmarks of aging: An expanding universe. Cell.

[33] Dasgupta N., Arnold R., Equey A., Gandhi A., Adams P.D. (2024). The role of the dynamic epigenetic landscape in senescence: Orchestrating SASP expression. NPJ Aging.

[34] Schmittgen T.D., Livak K.J. (2008). Analyzing real-time PCR data by the comparative C(T) method. Nat. Protoc..

[35] Xiong J., Wang F., Yang Y., Yang Y., Liu Z. (2024). Preventive effect of human umbilical cord mesenchymal stem cells on skin aging in rats. Heliyon.

[36] Zhang L., Pitcher L.E., Yousefzadeh M.J., Niedernhofer L.J., Robbins P.D., Zhu Y. (2022). Cellular senescence: A key therapeutic target in aging and diseases. J. Clin. Investig..

[37] Victorelli S., Lagnado A., Halim J., Moore W., Talbot D., Barrett K., Chapman J., Birch J., Ogrodnik M., Meves A. (2019). Senescent human melanocytes drive skin ageing via paracrine telomere dysfunction. EMBO J..

[38] Franco A.C., Aveleira C., Cavadas C. (2022). Skin senescence: Mechanisms and impact on whole-body aging. Trends Mol. Med..

[39] Zheng D.L., Wu Q.R., Zeng P., Li S.M., Cai Y.J., Chen S.Z., Luo X.S., Kuang S.J., Rao F., Lai Y.Y. (2022). Advanced glycation end products induce senescence of atrial myocytes and increase susceptibility of atrial fibrillation in diabetic mice. Aging Cell.

[40] Huang C.Y., Chen S.H., Lin T., Liao Y.W., Chang Y.C., Chen C.C., Yu C.C., Chen C.J. (2024). Resveratrol attenuates advanced glycation end product-induced senescence and inflammation in human gingival fibroblasts. J. Dent. Sci..

[41] Tarsounas M., Sung P. (2020). The antitumorigenic roles of BRCA1-BARD1 in DNA repair and replication. Nat. Rev. Mol. Cell Biol..

[42] Ghosh P., Fontanella R.A., Scisciola L., Pesapane A., Taktaz F., Franzese M., Puocci A., Ceriello A., Prattichizzo F., Rizzo M.R. (2023). Targeting redox imbalance in neurodegeneration: Characterizing the role of GLP-1 receptor agonists. Theranostics.

[43] Massacci G., Perfetto L., Sacco F. (2023). The Cyclin-dependent kinase 1: More than a cell cycle regulator. Br. J. Cancer.

[44] Radziszewski M., Galus R., Luszczynski K., Winiarski S., Wasowski D., Malejczyk J., Wlodarski P., Sciezynska A. (2024). The RAGE Pathway in Skin Pathology Development: A Comprehensive Review of Its Role and Therapeutic Potential. Int. J. Mol. Sci..

[45] Shu M., Cheng W., Jia X., Bai X., Zhao Y., Lu Y., Zhu L., Zhu Y., Wang L., Shu Y. (2023). AGEs promote atherosclerosis by increasing LDL transcytosis across endothelial cells via RAGE/NF-kappaB/Caveolin-1 pathway. Mol. Med..

[46] Sultana R., Parveen A., Kang M.C., Hong S.M., Kim S.Y. (2024). Glyoxal-derived advanced glycation end products (GO-AGEs) with UVB critically induce skin inflammaging: In vitro and in silico approaches. Sci. Rep..

[47] Xie J., Mendez J.D., Mendez-Valenzuela V., Aguilar-Hernandez M.M. (2013). Cellular signalling of the receptor for advanced glycation end products (RAGE). Cell. Signal..

[48] Chen M.C., Chen K.C., Chang G.C., Lin H., Wu C.C., Kao W.H., Teng C.J., Hsu S.L., Yang T.Y. (2020). RAGE acts as an oncogenic role and promotes the metastasis of human lung cancer. Cell Death Dis..

[49] Banerjee A., Singh P., Sheikh P.A., Kumar A., Koul V., Bhattacharyya J. (2024). Simultaneous regulation of AGE/RAGE signaling and MMP-9 expression by an immunomodulating hydrogel accelerates healing in diabetic wounds. Biomater. Adv..

[50] Pathomthongtaweechai N., Chutipongtanate S. (2020). AGE/RAGE signaling-mediated endoplasmic reticulum stress and future prospects in non-coding RNA therapeutics for diabetic nephropathy. Biomed. Pharmacother..

[51] Prasad K. (2019). AGE-RAGE stress: A changing landscape in pathology and treatment of Alzheimer’s disease. Mol. Cell. Biochem..

[52] Sarkar S. (2025). Pathological role of RAGE underlying progression of various diseases: Its potential as biomarker and therapeutic target. Naunyn-Schmiedeberg’s Arch. Pharmacol..

[53] Wei M., He X., Liu N., Deng H. (2024). Role of reactive oxygen species in ultraviolet-induced photodamage of the skin. Cell Div..

[54] Zhang S., Zhao X., Zhang W., Wei X., Chen X.L., Wang X. (2025). Zn-DHM nanozymes regulate metabolic and immune homeostasis for early diabetic wound therapy. Bioact. Mater..

[55] Huang J., Chen B., Wang H., Hu S., Yu X., Reilly J., He Z., You Y., Shu X. (2022). Dihydromyricetin Attenuates Depressive-like Behaviors in Mice by Inhibiting the AGE-RAGE Signaling Pathway. Cells.

[56] Wen X., Lv C., Zhou R., Wang Y., Zhou X., Qin S. (2024). The Molecular Mechanism Underlying the Therapeutic Effect of Dihydromyricetin on Type 2 Diabetes Mellitus Based on Network Pharmacology, Molecular Docking, and Transcriptomics. Foods.

[57] Amornsupak K., Thongchot S., Thinyakul C., Box C., Hedayat S., Thuwajit P., Eccles S.A., Thuwajit C. (2022). HMGB1 mediates invasion and PD-L1 expression through RAGE-PI3K/AKT signaling pathway in MDA-MB-231 breast cancer cells. BMC Cancer.

[58] Xie J., Liu J., Chen T.M., Lan Q., Zhang Q.Y., Liu B., Dai D., Zhang W.D., Hu L.P., Zhu R.Z. (2015). Dihydromyricetin alleviates carbon tetrachloride-induced acute liver injury via JNK-dependent mechanism in mice. World J. Gastroenterol..

[59] He C., Chen Y., Xie J., Luo M., Fisher D., Hien N.T.T., Musabaev E., Dang Y., Zhao L., Xia Y. (2024). Dihydromyricetin: An emerging compound with comprehensive effects on multiple systems. Front. Pharmacol..

[60] Fan L., Tong Q., Dong W., Yang G., Hou X., Xiong W., Shi C., Fang J., Wang W. (2017). Tissue Distribution, Excretion, and Metabolic Profile of Dihydromyricetin, a Flavonoid from Vine Tea (*Ampelopsis grossedentata*) after Oral Administration in Rats. J. Agric. Food Chem..

[61] Carry E., Kshatriya D., Silva J., Davies D.L., Yuan B., Wu Q., Patel H., Park E.R., Gilleran J., Hao L. (2021). Identification of Dihydromyricetin and Metabolites in Serum and Brain Associated with Acute Anti-Ethanol Intoxicating Effects in Mice. Int. J. Mol. Sci..

