# Supplementary material for: Dihydromyricetin May Attenuate Skin Aging as a RAGE Inhibitor
Source: Nutrients. 2025 May 29;17(11):1862. doi: 10.3390/nu17111862 (PMC12156997; doi:10.3390/nu17111862)
Supplement: Supplementary file 1 [file nutrients-17-01862-s001.zip › nutrients-3646488-supplementary.pdf]

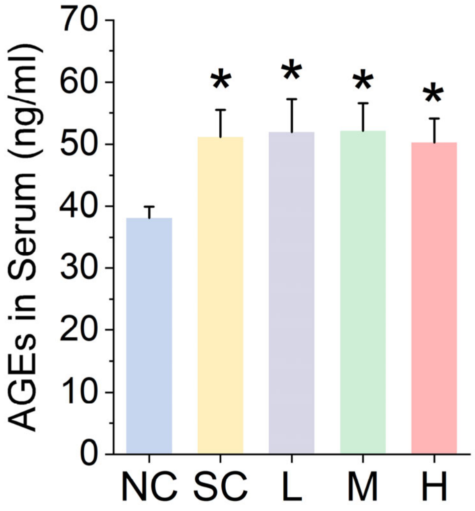

Figure S1. Levels of AGEs in rat serum. n=8; * p < 0.05 vs NC. Abbreviation for treatment groups: NC, normal control; SC, aging group treated with solvent solution; L, low-dose DHM; M, moderate-dose DHM; H, high-dose DHM.

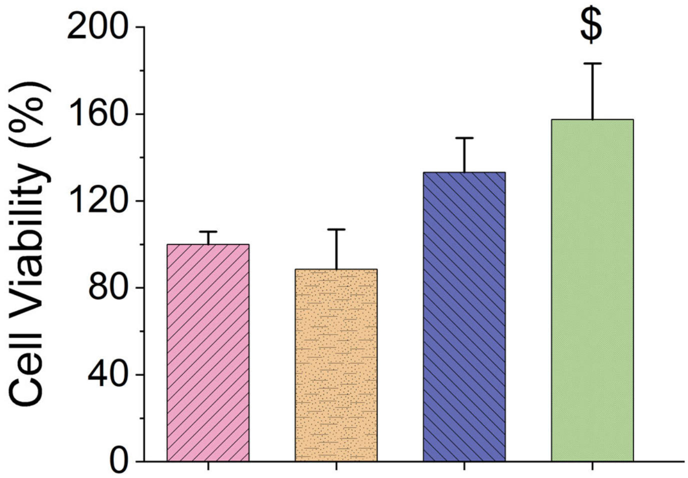

Figure S2. DHM promoted cell growth influenced by AGEs. Evaluation of cell viability when cells cultured with 50 μM DHM and 200 μg/mL AGEs for seven days, n=5. Values are shown as mean ± SEM. $ p < 0.05 vs AGEs

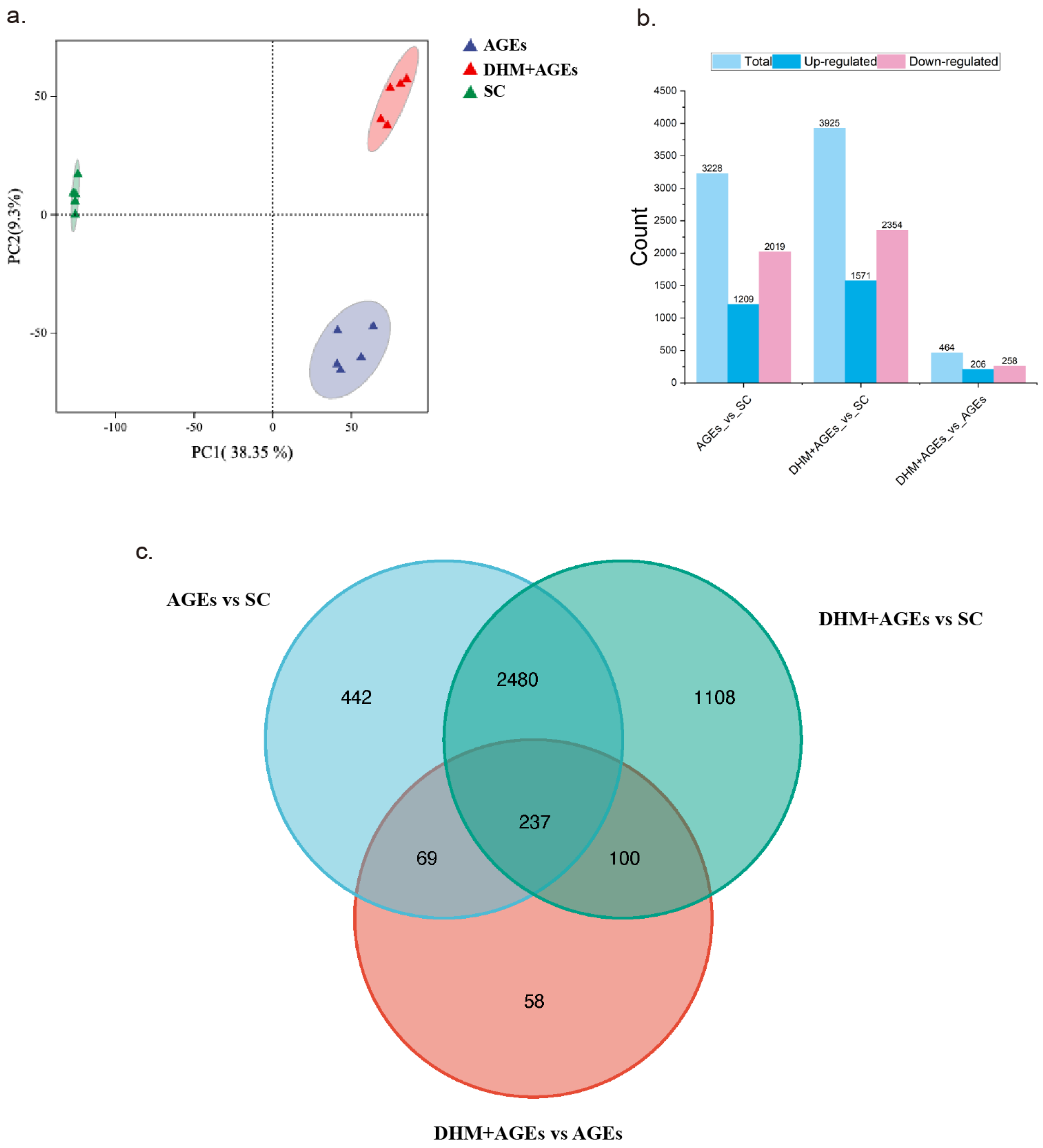

Figure S3. PCA analysis and difference in gene count across groups in transcriptomic analysis. (a) Plot of PCA analysis; (b) Comparison of gene count across groups; (c) Comparison of differentially expression gene count across groups.

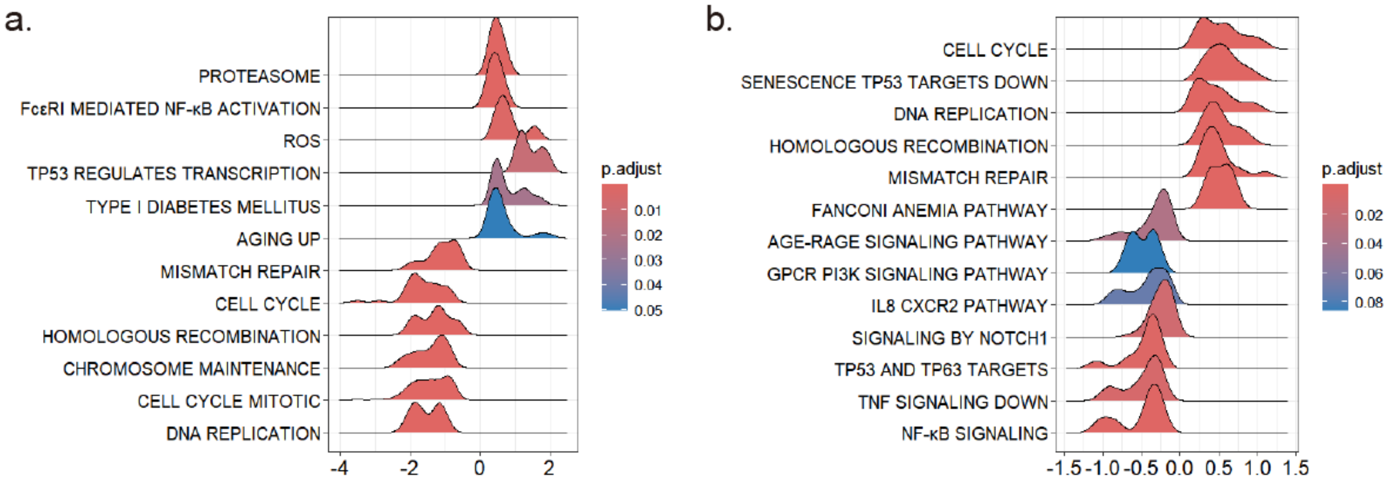

Figure S4. The ridgeline plot of Gene Set Enrichment Analysis (GSEA). (a) Pathways enriched by DEGs between non-senescent and senescent cells (treated with 200 μg/mL AGEs); (b) Pathways enriched by DEGs between cells treated with 16 μg/mL DHM and senescent cells. The plots displayed normalized enrichment score (NES) of representative enriched gene sets (*p* adjust <0.05) across groups; each ridge represented a gene set, with the x-axis showing NES values (positive values indicating upregulation, while negative values indicating downregulation) and the y-axis showing pathways.

Table S1. Level of COL1A1 in HFF-1 cells with different treatments (Mean ± SEM, *n* = 3)

Group	COL1A1 level (ng/mL, cultured for 24 h)	COL1A1 level (ng/mL, cultured for 48 h)
SC (with 0.025% DMSO)	7.57 ± 0.21	5.54 ± 0.13
200 μg/mL AGEs	6.77 ± 0.27	6.17 ± 0.38
12.5 μM DHM + 200 μg/mL AGEs	7.52 ± 0.66	6.25 ± 0.25
25 μM DHM + 200 μg/mL AGEs	6.72 ± 0.09	6.44 ± 0.35
50 μM DHM + 200 μg/mL AGEs	6.63 ± 0.32	6.51 ± 0.56
